# Is it time to consider depression as a major complication of type 2 diabetes? Evidence from a large population-based cohort study

**DOI:** 10.1007/s00592-021-01791-x

**Published:** 2021-09-08

**Authors:** Rossella Messina, Marica Iommi, Paola Rucci, Chiara Reno, Maria Pia Fantini, Carlotta Lunghi, Mattia Altini, Francesca Bravi, Simona Rosa, Antonio Nicolucci, Paolo Di Bartolo

**Affiliations:** 1 Department of Biomedical and Neuromotor Sciences, Alma Mater Studiorum-University of Bologna, Via San Giacomo, 12, 40126 Bologna, BO Italy; 2 Department of Health Sciences, Université du Québec À Rimouski, Rimouski, Canada; 3 Santé Des Populations Et Pratiques Optimales en Santé, Centre de Recherche du CHU de Québec, Québec, Canada; 4 Health Directorate, Romagna Local Health Authority, Bologna, Emilia-Romagna Region Italy; 5 Center for Outcomes Research and Clinical Epidemiology (CORESEARCH), Pescara, Italy; 6 Diabetes Unit, AUSL Romagna, Ravenna, Italy

**Keywords:** Complications, Clinical diabetes, Psychological aspects, Epidemiology

## Abstract

**Aims:**

Depression in type 2 diabetes may heavily affect the course of the disease. In this study, we investigated, among new cases with type 2 diabetes, the incidence and clinical predictors of depression and determined the extent to which depression constitutes a risk factor for acute and long-term diabetes complications and mortality.

**Methods:**

In this population-based retrospective cohort study, incident cases of type 2 diabetes without a prior history of depression were identified from the administrative databases of the Emilia-Romagna Region, Italy, between 2008 and 2017 and followed up until 2020. Logistic regression models were used to identify the predictors of depression. Cox regression models were used to estimate the risk of acute complications over three years, and the risk of long-term complications and mortality over ten years.

**Results:**

Incident cases with type 2 diabetes were 30,815, of whom 5146 (16.7%) developed depression. The predictors of depression onset were as follows: female sex, age > 65 years, living in rural areas and comorbid diseases. Depression in type 2 diabetes was associated with a 2.3-fold risk of developing acute complications, 1.6-fold risk of developing long-term complications and 2.8-fold mortality risk.

**Conclusions:**

Our findings highlight that depression is associated with an increased risk for complications in type 2 diabetes and mortality and should not be neglected. Therefore, it is important to promote screening activities and introduce targeted and personalized treatment for depression in order to reduce the risk of poor short- and long-term outcomes of diabetes.

**Supplementary Information:**

The online version contains supplementary material available at 10.1007/s00592-021-01791-x.

## Introduction

Type 2 diabetes, like other chronic conditions requiring intensive self-care management, is associated with high levels of distress, affecting the physical and psychological well-being and possibly leading to depression [1]. Indeed, depression is a common comorbidity among people with type 2 diabetes [2], and its incidence seems higher in the first year after glucose-lowering treatment initiation [3]. However, the relationship between type 2 diabetes and depression might be bidirectional, even if the underlying mechanisms are still unclear. Type 2 diabetes could lead to the development of depression due to the sense of loss of health and effectiveness, to behavioral and social factors [4], to biological factors such as insulin resistance, systemic inflammation, alterations in the hypothalamic–pituitary–adrenal axis [5]. At the same time, there is little evidence of a shared genetic vulnerability between depression and diabetes [5]. Despite good evidence supporting the role of diabetes as a trigger of the onset or worsening of depressive symptoms [6, 7], there is less convincing evidence that depression is a risk factor for the onset of type 2 diabetes. Depression may impact self-care and lifestyle behaviors, particularly related to diet and physical activity [8, 9], even if there is uncertain evidence that antidepressants reduce the risk of developing diabetes in normoglycemic individuals [5].

Moreover, clinical data on depression treatment suggest that improvement in depressive symptoms correlates with improved glycemic control in people with type 2 diabetes [5].

A significantly higher risk of developing depression in people with diabetes than in the general population has been reported [2], although the prevalence of depression in type 2 diabetes varies according to the assessment method [10]. In a systematic review conducted in 2019, almost one in four adults with type 2 diabetes experience depressive symptoms [7]. In a comprehensive meta-analysis of studies [11] where the diagnosis of depression was made using standardized diagnostic instruments, the prevalence of major depressive disorder in type 2 diabetes was estimated to be 14.5%, with an odds ratio of 1.73 for people with type 2 diabetes compared to the general population.

In people with diabetes, depression may negatively impact self-care [12], diabetes management [12], self-efficacy [13], cognitive outcomes [14], and medication adherence [15, 16], increasing the risk of developing diabetes complications [17, 18] and activating an additional vicious cycle [4]. Depression can affect all the aspects of quality of life, included sexual activity [19]. To this proposal, it is important to take into account depression and sexual dysfunctions in people with diabetes, as these mutually influence each other [20–22].

Several studies reported that depression or depressive symptoms in people with type 2 diabetes are associated with increased health care expenditure, cardiovascular diseases, and mortality [18, 23, 24].

A meta-analysis found that depression is associated with a 1.5-fold increased mortality risk in patients with diabetes [25]. However, the impact of depression on mortality varies among studies [18, 24–28], and it is still unclear whether the increased occurrence of diabetes complications drives this association [29]. Depression seems unrelated to microvascular complications or higher glycemia levels [30], while it has been linked to an increased risk for cardiovascular complications and all-cause mortality, but not with cardiovascular mortality or diabetes-related mortality [18]. On the other hand, a recent study from Quebec on individuals with type 2 diabetes newly treated with glucose-lowering drugs showed that depression was consistently associated with a higher risk of all-cause and cardiovascular-related mortality, regardless of the level of adherence to medications and age [27]. The excess mortality may be partially explained by the association between depression and the increased risk of cardiac events and cardiovascular-related mortality [26]. No evidence from the literature is available on the effect of depression on acute complications in type 2 diabetes.

One of the drawbacks of the studies investigating the association between diabetes and depression and the impact of depression on diabetes complications is that the temporal sequence of events is not taken into account. To address this limitation, we carried out a study based on administrative databases, in the attempt to determine the temporal sequence of depression and complications among new cases with type 2 diabetes.

Specifically, the aims of this study are (1) to estimate the incidence of depression over 10 years from the diagnosis of type 2 diabetes; (2) to identify the demographic and clinical predictors of depression; (3) to determine the extent to which depression constitutes a risk factor for acute and long-term complications of diabetes and mortality.

## Methods

### Setting and study population

In this population-based retrospective cohort study, the study population consisted of people with type 2 diabetes, aged 15 years or older, living in the Local Health Authority (LHA) of Romagna, that has a catchment area of about 1.1 million people.

Data for the present study were extracted from Emilia-Romagna administrative databases, including the Hospital Discharge Records (HDR) database; Mental Health Information System (MHIS); Residential Mental Health care (RMHC); Pharmaceutical databases; Regional mortality register. HDR database contains admissions and discharge dates, the primary and up to five secondary diagnoses and up to six interventions (identified using the International Classification of Diseases, Ninth Revision, Clinical Modification (ICD-9-CM coding system). MHIS database includes demographic characteristics and the ICD-9-CM diagnoses of all the adults who have at least one contact with the community mental health centers.

The RMHC database includes information on patients, discharged from no-profit or accredited private facilities, notably admission and discharge dates, principal diagnosis, and destination at discharge. The pharmaceutical databases include drugs reimbursed by the health care system and prescribed by the general practitioner or a specialist, or directly delivered by the hospital pharmacies. These databases contain information on the patient’s sex and age, prescriptions (substance name, ATC System code—V.2013, date of prescription filling, and number of packages), and prescribers. The regional mortality register database was used to collect the patient's date of death. These databases were linked through a unique anonymized patient identifier.

### Case definition of diabetes

Beneficiaries of the NHS aged 15 years or older and living in the LHA of Romagna were classified as patients with type 2 diabetes if they had at least one hospitalization with a primary or secondary diagnosis of diabetes (ICD-9-CM code 250.xx) and at least one prescription of Glucose-Lowering Medication (GLM) (ATC code A10), or at least three prescriptions of GLM in distinct periods during the follow-up.

To identify incident cases, we excluded all patients with at least one hospitalization or a GLM prescription in the three years before the date of entry into the study cohort. Uncertain cases of type 2 diabetes, such as patients with insulin as initial and unique treatment, and women diagnosed with gestational diabetes were excluded [31]. We further excluded patients with hospitalizations for the outcomes investigated (Supplementary Table 1) and patients with hospitalizations for depression or prescriptions of antidepressants in the three years before the diabetes diagnosis. The entry date into the study cohort was considered as the date of the diabetes diagnosis.

Patients were followed from the diagnosis of diabetes up to their death or October 31, 2020, whichever came first.

### Definition of depression

The presence of depression was ascertained using the following criteria: at least 1 prescription of antidepressant drugs (ATC code N06A), or at least 1 hospitalization (sources HDR, RMHD), or at least 1 outpatient service (source MHIS) with ICD-9-CM diagnosis codes for depression (see Supplementary Table 2). The first date of inpatient or outpatient record or antidepressant prescription was considered as the index depression date.

### Comorbid conditions

The presence of comorbid conditions in the three years preceding the onset of diabetes was determined for each patient. The comorbid conditions considered were as follows: other mental disorders (psychosis, bipolar disorders, anxiety/OCD, substance disorders), neurological disorders (epilepsy, dementia, Parkinson's disease), hypothyroidism, respiratory illness (COPD, asthma), and cancer (see Supplementary Table 3 for the detailed list of ICD-9-CM/ATC codes).

### Study outcomes

The primary outcome was the onset of acute diabetes complications (see Supplementary Table 1 for the detailed list of ICD-9-CM codes) in the first three years of follow-up.

Secondary outcomes were long-term diabetes complications and mortality within ten years from the onset of diabetes (see Supplementary Table 1 for the detailed list of ICD-9-CM codes). Complications were retrieved from the HDRs database.

### Statistical analysis

Demographic and clinical characteristics of patients who developed depression (Dep) during 10 years of follow-up and those who did not develop depression (Non-Dep) were summarized using absolute frequencies and percentages, means and standard deviations or medians and interquartile range (IQR), as appropriate.

Univariate logistic regression models were used to identify the predictors of depression. The possible predictors considered were sex, age (categorized as ≤ 35, 36–55, 56–65, 66–75, > 75 years), urbanization level of the municipality of residence, presence of comorbid conditions, and initial diabetes medication in the first month (1 oral GLM, 2 or more oral GLM, insulin, insulin + oral GLM).

Cox proportional-hazard models were used  to investigate whether depression was associated with complications and mortality, unadjusted and adjusted for confounders (sex, age group, presence of comorbid conditions, and initial diabetes medication). Patients were considered exposed to depression only if they developed depression before the outcomes or before the end of follow-up.

In the Cox regression models, depression was included as a time-dependent covariate to take into account that its onset could take place at different times during the follow-up. We tested the proportional-hazard assumption underlying these models using Schoenfeld residuals. When confounders did not meet the proportional-hazard assumption, they were used as strata of the baseline hazard. Results are expressed as odds ratios (ORs) (logistic regression) or hazard ratios (HRs) (Cox regression), with 95% confidence intervals (95%CI).

For all tests, significance was set as *p* < 0.05. Statistical analyses were performed using IBM SPSS version 25.0 and Stata 15.

## Results

**Fig. 1:**
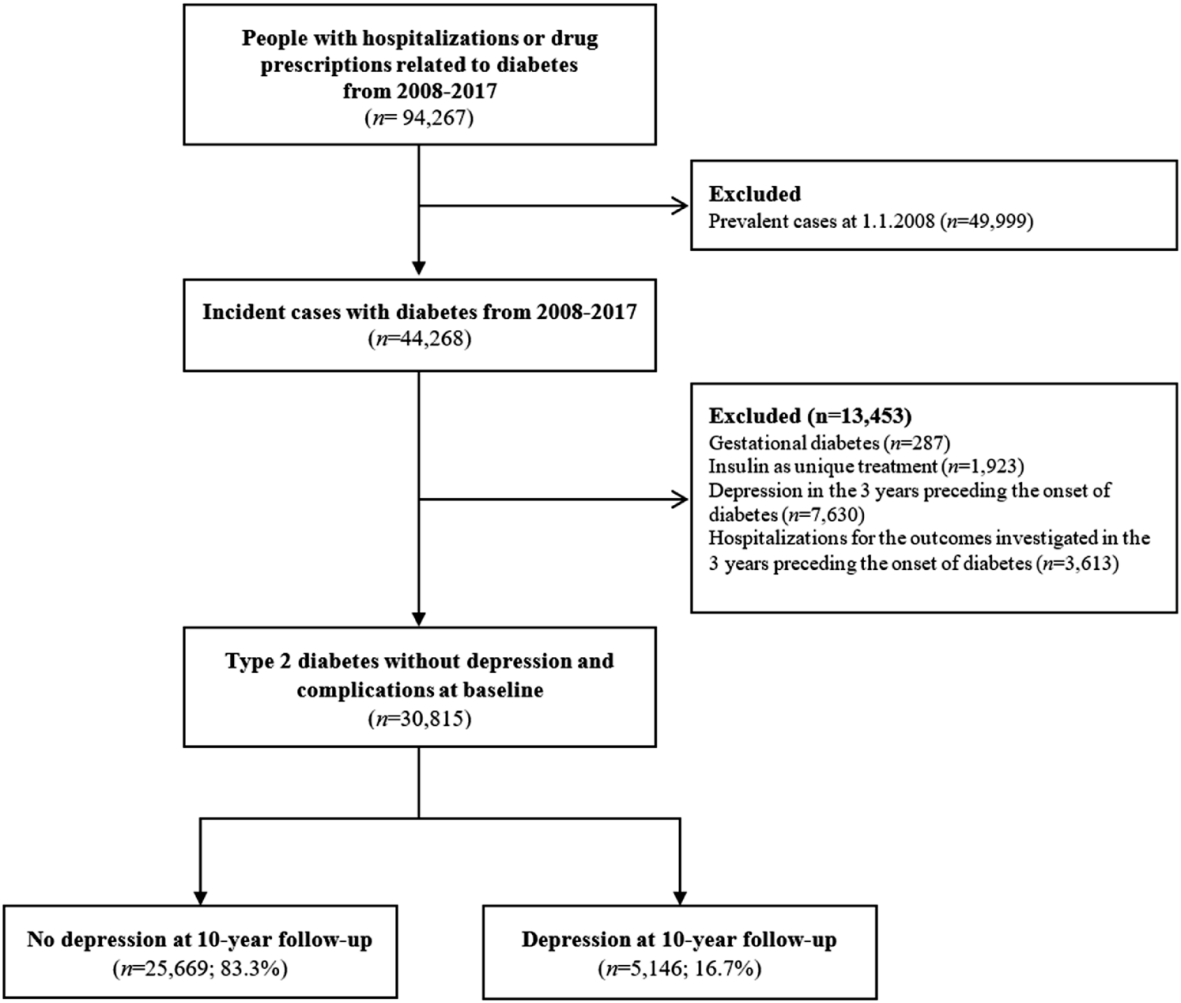
Flowchart of the study population

From January 1, 2008 to December 31, 2017, we identified 94,267 people with hospitalizations or drug prescriptions related to diabetes, of whom 44,268 were incident cases. After excluding 13,453 patients, the study cohort comprised 30,815 patients with type 2 diabetes (Fig. 1). During 10 years of follow-up, 5146 (16.7%) patients received a depression diagnosis or a prescription for an antidepressant drug after diabetes diagnosis (Dep group). Among patients in the Dep group, the onset of depression occurred on average 3.4 years after the diagnosis of diabetes (median = 2.8 years; IQR = 1.1 − 5.6). About 14.7% of the Dep group developed depression within six months from diabetes onset. Selective serotonin reuptake inhibitors (SSRIs) was the initial antidepressant therapy in 56.8% of patients with diabetes and depression, while 1.3% of patients with diabetes and depression did not receive antidepressant drugs (see Supplementary Table 4). The incidence of depression per 1000 person-years was 24.94.

### Demographic and clinical characteristics of patients with and without depression

Table 1 shows the baseline sociodemographic and clinical characteristics of the overall study population and the Dep and Non-Dep groups. Females were at a higher risk of depression than males (OR = 1.73; 95% CI [1.630; 1.839]). The age distribution differed between the two groups: compared to the age class 56–65, the older classes had a significantly higher risk of depression (66–75 age class: OR = 1.51, 95% CI [1.384; 1.642]; 75 + age class OR = 2.53, 95% CI [2.321; 2.764]). Patients living in rural areas were at higher risk of developing depression compared to patients living in high-density areas (OR = 1.15; 95% CI [1.051; 1.267]). Initial diabetes medication was unrelated to the development of depression.

**Table 1:** Sociodemographic and clinical characteristics of the study cohort and their association with depression

Sociodemographic and clinical characteristics	Total		Non-Dep		Dep		Univariate logistic regression models	
	(*n* = 30,815)		(*n* = 25,669)		(*n* = 5146)			
	*n*	%	*N*	%	*n*	%	*OR*	*95*% CI
*Sex*								
Female	13,444	43.6	10,615	41.4	2829	55.0	1.73	[1.630; 1.839]
Male	17,371	56.4	15,054	58.6	2317	45.0	Ref. cat.	
*Age class*								
≤ 35	1049	3.4	924	3.6	125	2.4	0.93	[0.759; 1.127]
36–55	7782	25.3	6790	26.5	992	19.3	1.00	[0.911; 1.096]
56–65	8332	27.0	7269	28.3	1063	20.7	Ref. cat.	
66–75	8072	26.2	6614	25.8	1458	28.3	1.51	[1.384; 1.642]
> 75	5580	18.1	4072	15.9	1508	29.3	2.53	[2.321; 2.764]
*Degree of urbanization*								
1–high-density area	11,715	38.0	9816	38.2	1899	36.9	Ref. cat.	
2–medium-density area	15,019	48.7	12,525	48.8	2494	48.5	1.02	[0.951; 1.084]
3–rural area	4081	13.2	3328	13.0	753	14.6	1.15	[1.051; 1.267]
*Initial diabetes medications*								
1 oral GLM	25,728	83.5	21,379	83.3	4349	84.5	Ref. cat.	
2 or more oral GLM	2462	8.0	2066	8.0	396	7.7	0.94	[0.842; 1.054]
Insulin	1214	3.9	1026	4.0	188	3.7	0.90	[0.768; 1.056]
Insulin + oral GLM	1411	4.6	1198	4.7	213	4.1	0.87	[0.753; 1.015]
*Comorbid conditions*								
Other mental disorders	386	1.3	283	1.1	103	2.0	1.83	[1.459; 2.300]
Neurological disorders	498	1.6	343	1.3	155	3.0	2.29	[1.892; 2.779]
Hypothyroidism	2332	7.6	1858	7.2	474	9.2	1.30	[1.170; 1.445]
Respiratory illness	2789	9.1	2206	8.6	583	11.3	1.36	[1.234; 1.497]
Cancer	1536	5.0	1233	4.8	303	5.9	1.24	[1.089; 1.411]

*GLM* Glucose-Lowering Medications; *Ref. cat.* reference category

Patients with comorbid conditions, like other mental disorders (OR = 1.83; 95% CI [1.459; 2.300]), neurological disorders (OR = 2.29; 95% CI [1.892; 2.779]), hypothyroidism (OR = 1.30; 95% CI [1.170; 1.445]), respiratory illness (OR = 1.36; 95% CI [1.234; 1.497]) and cancer (OR = 1.24; 95% CI [1.089; 1.411]), had a higher probability of developing depression.

### Depression and acute complications over three years

At three years, 162 patients (0.5%) experienced acute complications, after a median of 15.2 months. Of these, 18 (11.1%) had depression.

**Fig. 2:**
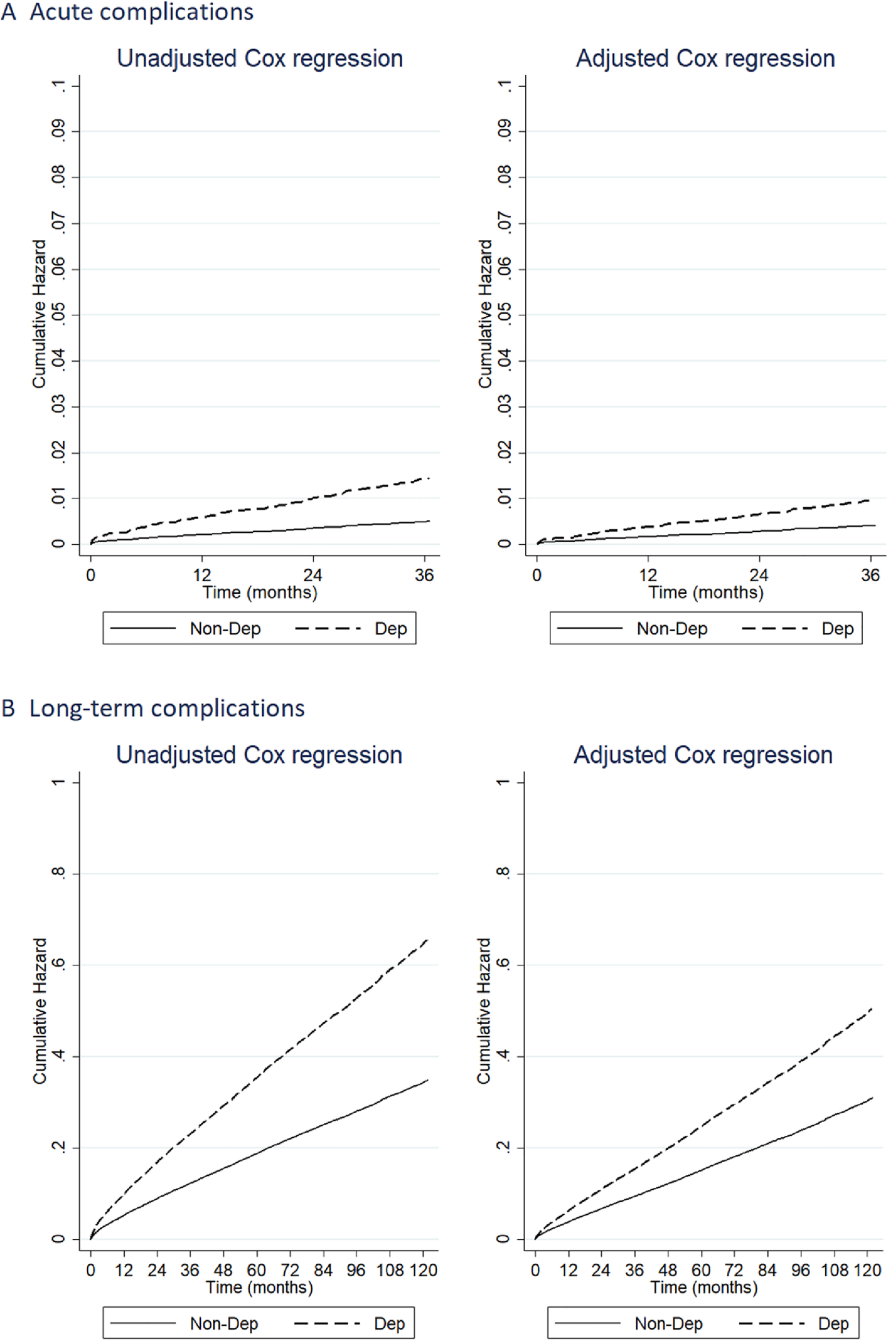
Acute (follow-up at three years, panel *A*) and long-term (follow-up at ten years, panel *B*) complications: cumulative hazard function from unadjusted and adjusted Cox regression models according to the presence of depression

Results from univariate Cox regression analysis indicate that depression was associated with an almost threefold risk of acute complications (HR = 2.88; 95% CI [1.745; 4.742]), and after adjusting for covariates the risk was only slightly attenuated (HR = 2.33; 95% CI [1.385; 3.921]). The cumulative hazard function by Dep and Non-Dep groups is shown in Fig. 2.

### Depression and long-term complications over ten years

Over the ten years of follow-up, long-term complications occurred in 7,488 patients (24.3%), on average follow-up of 76.3 months. Of these, 980 (13.1%) were diagnosed with depression.

In a univariate Cox regression model, patients with depression had a 1.9-time higher risk of long-term complications than patients without depression (HR = 1.89; 95% CI [1.760; 2.020]). In multiple Cox regression analyses, adjusted for age group, sex, initial diabetes medication, and comorbidities, depression was confirmed as an independent predictor of complications (HR = 1.64; 95% CI [1.523; 1.758]). The cumulative hazard functions for the Dep and Non-Dep groups are shown in Fig. 2.

### Depression and 10-year mortality risk

**Fig. 3:**
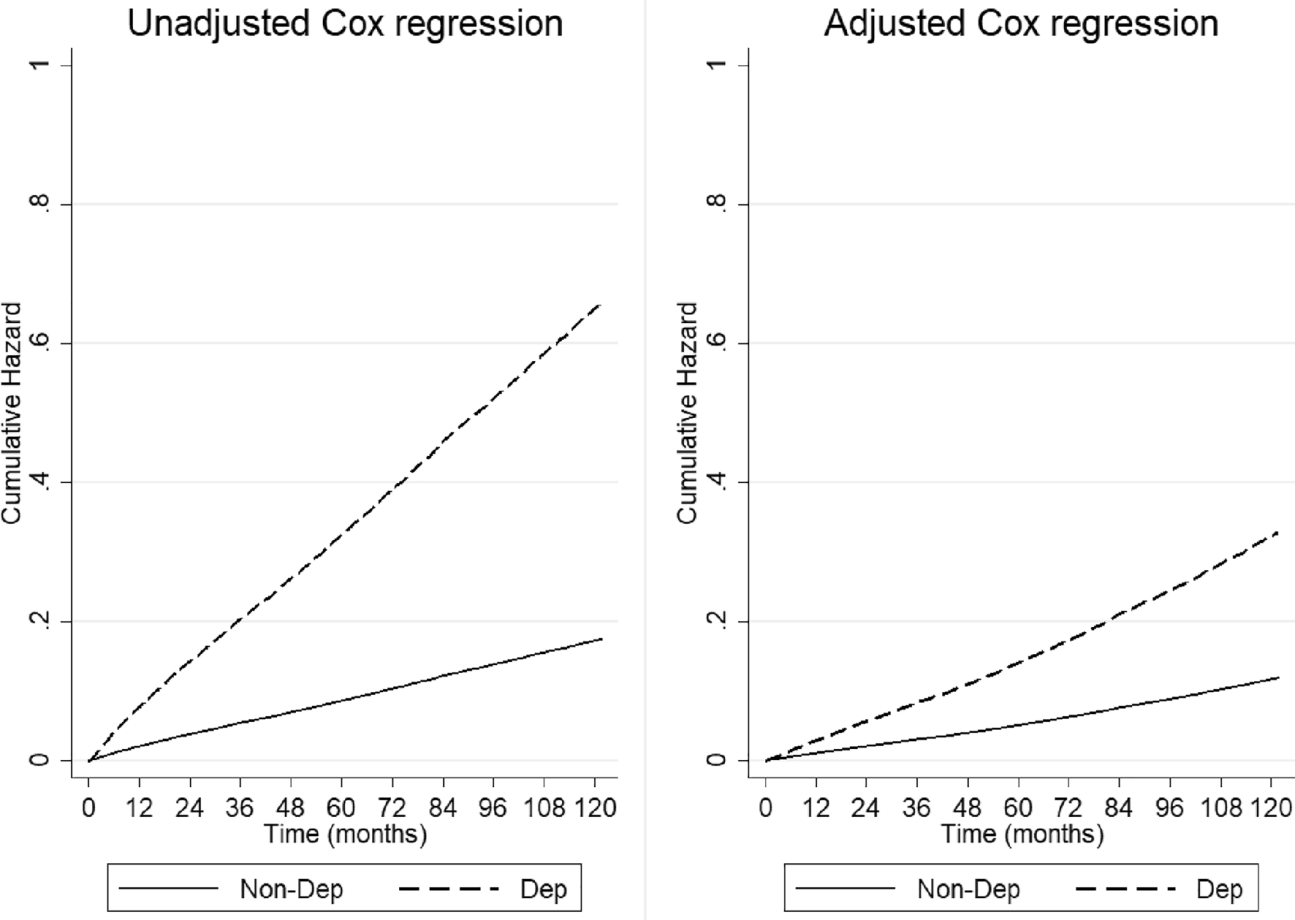
Ten-year all-cause mortality: cumulative hazard function from unadjusted and adjusted Cox regression models according to the presence of depression

Among the 5146 patients who developed depression, 1348 (26.2%) died during the ten years of follow-up, while in the same period, there were 3454 (13.5%) deaths in the Non-dep group. The median follow-up duration was 92.2 months (mean = 86.1 months). Cox regression analyses showed that depression was associated with a 3.8 (95% CI [3.539; 4.033]) mortality risk, that decreased to 2.8 in the adjusted model (95% CI [2.582; 2.962]). Figure 3 shows the cumulative hazard function for the Dep and Non-Dep groups.

## Discussion

The main results of this population-based retrospective cohort study are that a significant proportion of patients with type 2 diabetes and without a prior history of depression developed depression during follow-up and that depression negatively affects complications and mortality. We found that 16.7% of our population developed depression from 2008 to 2017, with on onset on average 3.6 years after diabetes diagnosis; the incidence of depression per 1000 person-years was 24.94. This figure was extremely high compared with those reported in other studies using administrative databases. In a study conducted in Quebec, using administrative data to assess the presence of depression, the authors reported a 12.61/1000 person-years incidence rate of depression during the first year after initiating a GLM, and an incidence of 9.47/1000 person-years in the eight-year study period [3]. Similar results were found in Saskatchewan, Canada [32] and Taiwan, China [33].

Our analyses indicate that female sex, age > 65 years, living in rural areas and comorbid conditions were associated with the occurrence of depression in type 2 diabetes.

A positive association between depression and female gender has been reported in other studies [3, 34]. Like a study carried out in Canada and based on claims data, we observed an increased risk of depression in people aged 65 years and more [3]. Nevertheless, another study found that lower age was associated with an increased risk for depression [35]. Consistent with (3), we observed that the presence of specific comorbidities, and especially psychiatric comorbidities or cancer, increased the risk of depression.

At variance with (3), we found a higher risk of depression in patients living in rural versus urban areas. Knowing the risk factors of depression in people with type 2 diabetes may help health care professionals identify timely patients at high risk, thus improving screening activities regarding the evaluation of psychological aspects and introducing targeted personalized treatment in diabetes care settings. As recommended by the standards of medical care in diabetes of the American Diabetes Association [36], the evaluation for depressive symptoms should be integrated into diabetes care as initial and annual screenings, and, as suggested by the PsychoSocial Aspects of Diabetes study group of the European Association for the Study of Diabetes, person-centered outcomes should be used longitudinally and integrated into diabetes registers for clinical management and risk stratification [37].

The key findings of our study are that depression highly increased the risk of developing acute complications over three years (by 2.33 times) and the risk of developing long-term complications over ten years (by 1.6 times) above and beyond demographic characteristics, initial treatment and comorbid conditions.

Evidence from the literature suggests people with type 2 diabetes have a twofold higher risk than the general population of fatal and non-fatal coronary heart disease, hemorrhagic or ischemic stroke [38]. Diabetes cardiovascular complications are considered major complications, that not only negatively affect the global health, but also the quality of life of people with diabetes [39], increasing the burden of the disease in terms of health care utilization [40], economic expenditure [41], and mortality.

Moreover, in our study, we found that 26.2% of people with depression versus 13.5% without depression died during the ten-year follow-up.

Our findings suggest that people with type 2 diabetes and subsequent depression should be protected from the increased risk of acute complications possibly caused by depression itself. Therefore, it is important to avoid, when possible, complex therapeutic regimens and the use of pharmacological treatments characterized by a high risk of hypoglycemia. Adherence to therapy and a regular glucose control assessment should be promoted for these patients by involving the family or by activating home care support systems (multidisciplinary taking and social network). Our findings highlight the significant impact of depression and suggest that it should be considered a major complication of type 2 diabetes and a mediator of poor outcomes. The presence of depression should be evaluated, especially in older people, as often as the presence of other major complications. In case of depression, a comprehensive medical evaluation and approach should be assured to these patients, given their high level of frailty. Furthermore, introducing new practices among health care professionals to take into account patients’ emotional needs may enhance health care professionals’ efforts to address psychological health in adults with diabetes [42].

### Strengths and limitations

As for other studies based on administrative databases, this study has some limitations. One intrinsic limitation is the lack of clinical information that could allow a better characterization of patients and the identification of clinical predictors of poor outcomes, because administrative databases are collected for purposes different from research. Moreover, the case definition for depression is based on antidepressant prescriptions and information from hospitalizations and outpatient services; we were not able to confirm the diagnosis of depression via clinical interviews or questionnaires evaluating depressive symptoms. Furthermore, we are aware that antidepressant prescriptions are often used as medication for many other clinical conditions, such as eating disorders, sleep disorders, premature ejaculation and chronic pain. Patients not seeking treatment for depression or those receiving psychological therapies in private practices may therefore have been missed, which could have led to an underestimation of the actual risk of complications and mortality. Another limitation is that diabetes complications were collected from hospital discharge records; therefore, complications managed in outpatient settings were not identified.

Nevertheless, this study also has several strengths. The main strength consists of its longitudinal design. Indeed, we were able to model in our statistical analysis the temporal sequence of depression and complications in this large cohort of people newly diagnosed with type 2 diabetes. Using time-dependent Cox analyses, we avoided immortal-time bias and estimated the adjusted effect of depression on the onset time of complications and death. In this way, we corroborated with empirical evidence the hypothesis of a causal relationship between depression and diabetes outcomes. In addition, the use of a population-based cohort minimized the risk of selection bias. Administrative databases of Emilia-Romagna region proved to be high-quality and precious data sources for epidemiological studies [43, 44].

## Conclusion

Our study confirms highlights that depression is associated with an increased risk for chronic diabetes complications and all-cause mortality in patients with diabetes but provides new evidence of the impact of depression on acute complications. For this reason, it is important to help health care professionals identify timely patients at high risk of developing depression through screening activities and, when depression is ascertained, introduce targeted and personalized treatment in diabetes health care pathways. Evidence supports the effectiveness of psychosocial interventions, antidepressant medication and collaborative care in the treatment of depression in patients with diabetes [45].

Future research is needed to evaluate the impact on acute and long-term complications and mortality of an intensive depression screening and comprehensive treatment in people with type 2 diabetes.

## Supplementary Information

Below is the link to the electronic supplementary material.

Supplementary file1 (DOCX 27 KB)

## Data Availability

The data sets generated during and/or analyzed during this study are available from the corresponding author on reasonable request.
