# Supplementary material for: Is it time to consider depression as a major complication of type 2 diabetes? Evidence from a large population-based cohort study
Source: Acta Diabetol. 2021 Sep 8;59(1):95–104. doi: 10.1007/s00592-021-01791-x (PMC8758621; doi:10.1007/s00592-021-01791-x)
Supplement: Supplementary file 1 — Supplementary file1 (DOCX 27 KB) [file 592_2021_1791_MOESM1_ESM.docx]

**Supplementary Table 1**. ICD-9-CM codes of acute and long-term diabetes complications.

| **Diabetes complications** | **ICD-9-CM diagnosis and surgery procedure codes** |
| --- | --- |
| **Acute** | coma (250.3, 251.0)  hyperosmolarity (250.2)  hypoglycaemia (251.2)  ketoacidosis (250.1, 276.2) |
| **Long-term** | |
| *Cardio-Cerebrovascular* | acute myocardial infarction (410)  cerebrovascular disease (433, 435-437)  diabetes circulatory complications (250.7)  gangrene (785.4)  hischemic/emorragic stroke (430-432, 434)  hypertension (402.01; 402.11; 402.91; 404.01; 404.11; 404.91)  ischemic heart disease (411-414)  other hearth disease (428, 429.1)  peripheral artery disease, (440.2; 440.3; 443.81)  ulcers (707.1) |
| *Neuropathy* | disorders of the peripheral nervous system (354-355, 357.2)  neuropathy (350; 351; 378.51; 378.52; 378.53; 378.54)  peripheral autonomic neuropathy (337.1) |
| *Renal* | acute renal kidney (584)  chronic renal disease, nephritic syndrome (585, 581.81)  diabetes renal complications (250.4)  dialysis (V45.1; V56.1; V56.2; V56.3) |
| *Ophthalmic* | diabetes ophthalmic complications (250.5)  disorders of the eye and adnexa (362.0; 362.01; 362.02; 362.55, 364.42, 365.63, 369)  maculopathy (362.07) |
| *Amputations* | surgery procedure code (84.11; 84.12; 84.13; 84.15; 84.17) |
| *Diabetes with other specified or unspecified complications* | diabetes with other specified manifestations (250.8)  diabetes with unspecified complication (250.9) |

**Supplementary Table 2**. ICD-9-CM codes of depression.

| **ICD-9-CM diagnosis codes of depression** | |
| --- | --- |
| **Depression** | 296.2, 296.20, 296.21, 296.22, 296.23, 296.24, 296.25, 296.26, 296.3, 296.30, 296.31, 296.32, 296.33, 296.34, 296.35, 296.36, 296.9, 296.90, 296.99, 300.4, 309.0, 309.1, 311. |

**Supplementary Table 3**. ICD-9-CM and ATC codes of comorbid conditions.

| **Comorbidity** | **ICD-9 CM and ATC codes** |
| --- | --- |
| **Other**  **mental disorders** | **ATC**  Anxiety/OCD (N05B), Bipolar disorders (N05AN), Psychosis (N05A) excluding (N05AN), Addictive Disorders (N07B)  **ICD-9 CM**  Psychoses (293.8, 295, 296.04, 296.14, 296.44, 296.54, 297, 298), Drug Abuse (292, 304, 305.2, 305.3, 305.4, 305.5, 305.6, 305.7, 305.8, 305.9, V65.42), Alcohol Abuse (265.2, 291.1, 291.2, 291.3, 291.5, 291.8, 291.9, 303.0, 303.9, 305.0, 357.5, 425.5, 535.3, 571.0, 571.1, 571.2, 571.3, 980, V11.3) |
| **Neurological disorders** | **ATC**  Epilepsy (N03A, N05CD08) excluding (N03AA02, N03AE01, N03AF01, N03AG02, N03AX09, N03AX12, N03AX16, N03AX21), Dementia (N06D, N06BX13), Parkinson disease (N04) excluding (N04BC01)  **ICD-9 CM**  Dementia (290, 290.0, 290.1, 290.10, 290.11, 290.12, 290.13, 290.2, 290.20, 290.21, 290.3, 290.4, 290.40, 290.41, 290.42, 290.43, 290.8, 290.9, 294.1, 294.10, 294.11, 331.2, 331.0, 293.0, 293.1, 293.9, 294.0, 294.8, 294.9, 310.0, 310.1, 310.2, 310.8, 310.9), Other Neurological Disorders (331.9, 332.0, 332.1, 333.4, 333.5, 333.92, 334, 335, 3362, 340, 341, 345, 348.1, 348.3, 780.3, 784.3) |
| **Respiratory illness** | **ATC**  R03  **ICD-9 CM**  416.8, 416.9, 490, 491, 492, 493, 494, 495, 496, 500, 501, 502, 503, 504, 505, 506.4, 508.1, 508.8 |
| **Hypothyroidism** | **ATC**  H03A  **ICD-9 CM**  240.9, 243, 244, 246.1, 246.8 |
| **Cancer** | **ATC**  H01CB, L01, L02, L03AC, L03AX, L04AX02, L04AX04, L04AX06, V03AF excluding H01CB01, L01AA01, L01DB07, L01XC02, L01XE31, L01XX05, L01XX14, L02AB01, L03AX13  **ICD-9 CM**  Lymphoma (200, 201, 202, 203.0, 238.6), Metastatic Cancer (196, 197, 198, 199), Solid Tumor without Metastasis (140, 141, 142, 143, 144, 145, 146, 147, 148, 149, 150, 151, 152, 153, 154, 155, 156, 157, 158, 159, 160, 161, 162, 163, 164, 165, 166, 167, 168, 169, 170, 171, 172, 174, 175, 176, 177, 178, 179, 180, 181, 182, 183, 184, 185, 186, 187, 188, 189, 190, 191, 192, 193, 194, 195) |

**Supplementary Table 4.** Initial antidepressant therapy of patients with diabetes and depression (n=5,146).

| **Classes of antidepressant drugs** | **n** | **%** |
| --- | --- | --- |
| No antidepressant drugs | 68 | 1.3% |
| Tricyclic antidepressants (TCAs) | 491 | 9.5% |
| Selective serotonin reuptake inhibitors (SSRIs) | 2923 | 56.8% |
| Serotonin–norepinephrine reuptake inhibitors (SNRIs) | 533 | 10.4% |
| Other antidepressant drugs | 1131 | 22.0% |
